# Resting Blood Pressure in Master Athletes: Immune from Hypertension?

**DOI:** 10.3390/sports11040085

**Published:** 2023-04-18

**Authors:** Mike Climstein, Joe Walsh, Mark DeBeliso, Tim Heazlewood, Trish Sevene, Luke Del Vecchio, Kent Adams

**Affiliations:** 1 Physical Activity, Sport and Exercise Research (PASER) Theme, Faculty of Health, Southern Cross University, Bilinga, QLD 4225, Australia; 2 Physical Activity, Lifestyle, Ageing and Wellbeing, Faculty Research Group, Faculty of Health Sciences, The University of Sydney, Camperdown, NSW 2050, Australia; 3 Sport Science Institute, Sydney, NSW 2000, Australia; 4 Department of Kinesiology and Outdoor Recreation, Southern Utah University, Cedar City, UT 84720, USA; 5 Kinesiology Department, California State University Monterey Bay, Seaside, CA 93955, USA; 6 Faculty of Health Sciences, Southern Cross University, Bilinga, QLD 4225, Australia

**Keywords:** resting blood pressure, veteran athletes, exercise, risk factors, physical activity, sports medicine, chronic disease, health, aged

## Abstract

Ageing is associated with decreased physical activity, obesity and increased risk of hypertension (HTN). Master athletes (MA) have either pursued a physically active lifestyle throughout their life or initiated exercise or sport later in life. We assessed resting blood pressure (BP) in male and female World Masters Games (WMG) athletes. This was a cross-sectional, observational study which utilized an online survey to assess the blood pressure (BP) and other physiological parameters. Results: a total of 2793 participants were involved in this study. Key findings included differences between genders with males reporting higher resting SBP (+9.4%, *p* < 0.001), resting DBP (+5.9%, *p* < 0.001) and mean arterial pressure (+6.2%, *p* < 0.001). Significant differences (*p* < 0.001) were also identified when comparing WMG athletes’ resting BP results (genders combined) to the general Australian population with WMG athletes having a lower SBP (*p* < 0.001, −8.4%) and DBP (*p* < 0.001, −3.6%). Additionally, 19.9% of males and 49.7% of female WMG participants were normotensive whereas 35.7% of the general Australian population were normotensive. Only 8.1% of the WMG athletes (genders combined) were found to be HTN compared to 17.2% in the general Australian population. These findings reflect a low prevalence of HTN in WMG participants and support our hypothesis of a low prevalence of HTN in an active, but aged cohort of MA.

## 1. Introduction 

Cardiovascular disease is a group of disorders of the heart and blood vessels which includes, for example, coronary heart disease and cerebrovascular disease. Furthermore, hypertension is also linked to a wider range of cardiovascular disease disorders which includes fatal and non-fatal cardiovascular disease (i.e., acute myocardial infarction) as well as congestive heart failure, peripheral arterial disease, stable angina and kidney disease [1]. Cardiovascular disease remains the leading cause of death in the world with an estimated 18 million lives lost each year to this disease [2]. In Australia, it has been estimated that 27% of all deaths were attributed to cardiovascular disease and an additional one million Australians are currently living with the disorder [3]. The Centers for Disease Control and Prevention [4] lists a number of risk factors for the development of coronary heart disease and they include hypercholesterolemia, diabetes (type 1 and type 2), smoking, obesity, unhealthy diet, physical inactivity and hypertension. The latter, hypertension, also referred to as systemic arterial hypertension, has been defined as a resting elevated blood pressure, above a typical threshold (i.e., ≥130/80 mHg) [5], and this is used to diagnose and treat hypertension. Hypertension has been associated with the strongest causal evidence and recognized as a predominant risk factor for the causation of cardiovascular disease [1,6,7,8]. Hypertension is a common medical condition with an estimated 1.3 billion adults, aged 30 to 79 years, suffering from the condition worldwide [9]. Although hypertension is highly prevalent, especially with increasing age, it is quantitatively the most important modifiable risk factor for the development of cardiovascular disease [10]. As a modifiable risk factor, hypertension can be modified by lifestyle changes such as a low sodium diet (<920 mg/d). A low sodium diet has been shown to significantly reduce resting blood pressure in hypertensive and normotensive individuals [11], and sodium restriction was shown to have a positive linear relationship in both systolic blood pressure (SBP) and diastolic blood pressure (DBP) [12]. As sodium intake was reduced, there were greater improvements in the reductions seen in both SBP and DBP. Tsai and colleagues [13] reported that cessation of smoking was associated with a decrease in both SBP (−5.0 mmHg) and DBP (−3.1 mmHg). 

Physical activity and exercise are another lifestyle management strategy for prevention and treatment of hypertension [14]. For example, physical activity and exercise lowers blood pressure (SBP and DBP) by improving endothelial function and reducing peripheral resistance [15]. Additionally, the anti-hypertensive effects of exercise are mediated through enhanced baroreceptor sensitivity, reduced peripheral vascular resistance and a vasodilator effect due to increased nitric oxide [15]. Dimeo et al. [16] evaluated the effectiveness of aerobic exercise in blood pressure resistant hypertensives. They reported that 8 to 12 weeks of treadmill exercise in patients with resistant hypertension significantly (*p* = 0.03) reduced their resting SBP (−6 mmHg ± 12) and DBP (−3 mmHg ± 7). de Barcelos et al. [17] completed a systematic review (*n* = 24, 1207 participants) on the effects of aerobic training, with and without exercise progression. They found a significant improvement in SBP in both the group with progression (−10.67 mmHg) and the group without progression (−10.17 mmHg). They also found significant improvements in DBP with (−5.49 mmHg) and without (−6.51 mmHg) exercise progression. 

Lemes and colleagues [18] completed a systematic review and meta-analysis of randomized controlled studies (*n* = 8), and they reported that long-term resistance training significantly reduced SBP by (4.1 mmHg), (95% CI 0.55 to 7.14), and seven studies reported a negligible improvement in DBP (−1.4 mmHg), (95% CI −0.19, 2.98; *p* = 0.08). Boeno and colleagues investigated the effects of 12 weeks of supervised, combined aerobic and resistance training in middle-aged (30 to 59 years) individuals with hypertension. They reported the combined training resulted in a significant reduction in resting SBP (−7.2 mmHg ± 7.9) and DBP (−4.4 mmHg ± 5.8). Given the established benefits of both aerobic exercise, resistance training exercise (with and without progression) and combined aerobic and resistance training exercise on blood pressure, it is rational to assume that individuals who participate in physical activity and/or exercise in middle and older ages would display an improved resting blood pressure as compared to the general population. 

Master athlete is a term applied to individuals (male and female), typically aged 35 years and older, who exercise and compete on a regular basis in organized sports competitions with similar-aged individuals [19]. There is no specific age which defines a master athlete as different sporting organizations define a master athlete with different minimum ages. For example, the Federation Internationale De Natation defines master athletes as those who compete in swimming who are a minimum of 25 years of age [20]. In contrast, the USA Track and Field organization defines the minimum age for master athletes as 35 years of age and older [21]. The World Masters Games (WMG) is an international sporting competition for master athletes which is conducted every four years. The WMG is the largest event of its kind, far exceeding participation at the Olympics (i.e., 2022 Olympics had 2871 participants, 650 para-Olympic athletes). For example, the Australian (2009) and New Zealand (2017) WMG had over 28,500 master athletes competing in 28 different sports. 

Master athletes have been reported to be significantly healthier than the general population in a number of health outcomes [22], including numerous chronic diseases such as asthma, coronary heart disease, stroke, cancer (all types combined), depression, diabetes (type 1 diabetes mellitus, type 2 diabetes mellitus), hypercholesterolemia, hypothyroidism, osteoporosis, Parkinson’s’ disease and peripheral arterial disease [23]. Although the available published literature on master athletes is limited, there are published studies which have identified health benefits in biomedical health determinants and risk factors including lower risk of obesity, as assessed by body mass index (BMI) [19,24,25,26,27,28,29,30], lipids [31] and more recently fasting plasma glucose [32]. There have been very limited studies investigating blood pressure and hypertension in master athletes; however, they have included smaller sample sizes (*n* = 214 and *n* = 264) and are limited to only male participants. [33,34]. Given the increasing prevalence of hypertension with ageing and the recognized benefit of exercise (aerobic and resistance) on reductions in blood pressure, we believed that master athletes would be an ideal cohort to investigate resting blood pressure and the prevalence of hypertension. Given the previous beneficial health outcomes reported in master athletes (BMI, lipids, glycemia), they have subsequently been referred to as a model of successful ageing [35]. We therefore chose to investigate another health determinant, resting SBP and DBP, in a large sample of male and female master athletes who participated in the WMG. The categories of SBP and DBP as well as resting SBP and DBP were our primary outcome variables. We hypothesized that master athletes would have a lower prevalence of hypertension and mean lower resting SBP and DBP as compared to the general populations.

## 2. Materials and Methods

We utilized the Strengthening the Reporting of Observational Studies in Epidemiology (STROBE, Supplementary Materials) checklist (cross-sectional studies) [36] guidelines in the preparation of this manuscript.

### 2.1. Ethics Approval and Survey

This epidemiological study employed a cross-sectional, observational survey design which investigated the demographics, cardiometabolic risk factors and resting SBP and DBP of participants at the WMG. Ethics approval to conduct this study was in accordance with the ethics standards (revised in 2008) by the Helsinki declaration [37]. Bond University’s Human Research Ethics committee approved this study and subsequently granted ethical approval (BUHREC RO1682). This study was also approved by the World Masters Games Organizing Committee; however, the committee stipulated that the survey must be delivered only online to minimize any potential disruption to the WMG participants while in preparation or while competing in the WMG. The survey was open prior to the start of the WMG, during the WMG and following the WMG for a total of four months.

Accordingly, we designed an online, cloud-based survey using an open-source application (LimeSurvey, available under the GNU general public license) to distribute the survey to a convenience sample of WMG MA participants. To facilitate participation, we utilized questions in the following format to expedite completion of the survey: array, drop-down choices, single choice, multiple choice and numerical input. We also employed filters and single/multiple tick wherever possible to expedite completion of the survey as a strategy to increase participation [38]. 

All WMG participants were eligible to participate in this study. Electronic invitations were sent to all WMG athletes who provided a valid email address (*n* = 24,578) when they registered for their sport(s) to compete in at the WMG. There was a total of 28,676 competitors at the games participating in at least one of the 43 sports. Participants were informed in the invitation (and at the beginning of the survey, prior to informed consent section) that they needed to obtain information from their primary care physician or medical specialist which included height, mass, waist circumference and resting blood pressure. We have previously published the findings about body mass index (BMI) [19], obesity and fasting blood glucose [32] in master athletes. Due to confidentiality and ethical concerns, participants were not required to upload their medical reports, which may have contained other personal medical results or findings not relevant to this study. Once a participant agreed to participate in the study by completing the informed consent, they were allowed access to the survey questions.

There were three main sections of the survey. The sections included participant demographics, participants’ medical health history and physiological outcome variables which included resting systolic and diastolic blood pressure. 

### 2.2. Biometrics Classifications

We utilized the Royal Australian College of General Practitioners (RACGP) [39] classification for BMI in our WMG participants where an underweight classification of BMI was calculated as <18.5 kg/m^2^, normal BMI was calculated as ≥18.5 to <25 kg/m^2^, overweight was calculated as a BMI of ≥25.0 to <30 kg/m^2^ and obese was calculated as a BMI ≥ 30.0 kg/m^2^. We also used the RACGP classifications [39], gender specific, for waist circumference. For males and females, a normal waist circumference as <94 cm and <80 cm, respectively. An increased risk of obesity-related conditions was a waist circumference of ≥94 cm to <102 cm for males and ≥80 cm to <88.0 cm for females [40]. A waist circumference associated with a very high risk of obesity-related complications was ≥102 cm in males and ≥88 cm in female WMG participants.

In 2017, the American College of Cardiology in conjunction with the American Heart Association [5,41] released new blood pressure guidelines for categorizing hypertension. We utilized these more conservative categories in our study for categorizing blood pressure as they are now used widely in clinical practice. A normal classification for SBP and DBP was a reading of <120 mmHg and <80 mmHg, respectively. An elevated blood pressure classification was a SBP and DBP of ≥120 to <130 mmHg and <80 mmHg, respectively. Regarding hypertension (Stage 1), this was classified as a SBP from ≥130 to <140 mmHg or DBP ≥ 80 to <90 mmHg. Hypertension (Stage 2) was a SBP ≥ 140 mmHg or DBP ≥ 90 mmHg.

### 2.3. National Comparative Data

As our data set of participants was large and the majority (79.1%) of participants were from Australia, we chose to use the Australian Bureau of Statistics Health Survey (*n* approx. 11,000) BP results for a comparative group. We also attained comparative BP data from the 2017–2018 National Health and Nutrition Examination Survey (NHANES, *n* = 4689 aged 18 years and older) as a second comparative group [42].

### 2.4. Statistical Analyses

Descriptive statistics included mean ± standard deviation (SD) and were calculated for physiological variables which included age, mass, BMI, waist circumference and resting blood pressure (SBP and DBP). Normality of the data was assessed by kurtosis, skewness, Q-Q plots and the Kolmogorov–Smirnov test (with Lilliefors significance correction) [43]. Significance between genders was determined using an independent samples *t*-test. An ANOVA was used to assess statistical significance with SBP and DBP when age was stratified by decades. Significance between genders combined and the general Australian population was assessed using a one-sample *t*-test. Bivariate correlations (2-tailed) were analyzed on selected outcome variables. Alpha was set a priori at *p* < 0.05 to determine statistical significance. All data analyses were completed using SPSS (Ver 28.0.0.0 (190); SPSS Inc., IBM Company, Armonk, NY, USA).

## 3. Results

### 3.1. Study Participants

A total of 26,676 WMG participants registered with a valid email address to participate in the WMG. Once cleansed (bounced, spam, unspecified), a total of 24,528 email invitations for the survey were sent to WMG participants, of which 2793 (11.4%) provided the necessary biometric data which included both resting SBP and DBP. 

**Figure 1:**
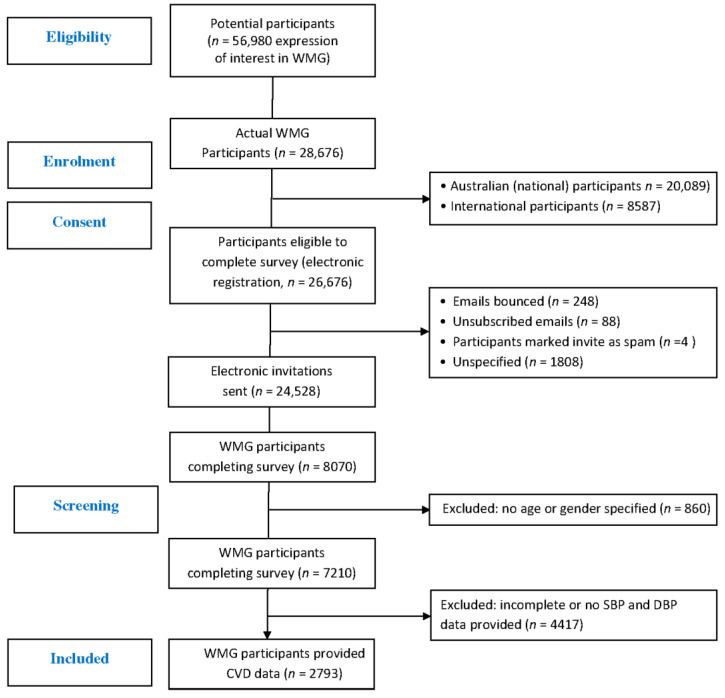
CONSORT flow diagram of WMG participants.

**Figure 2:**
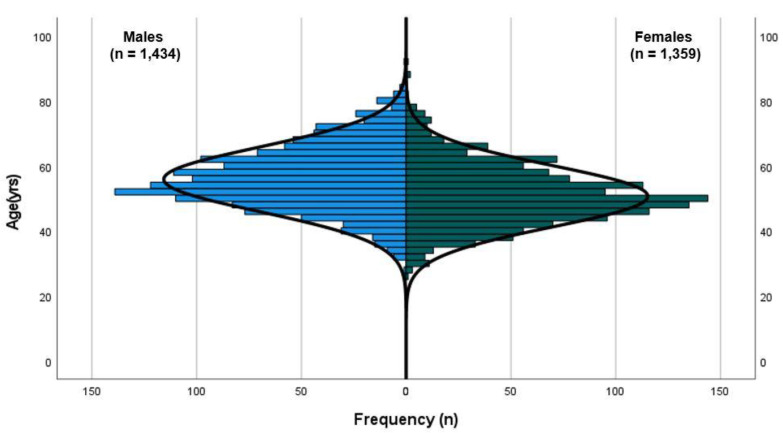
Population pyramid of WMG participants (line of normality illustrated).

These participants were included in this study for analyses between genders and population norms (Figure 1). Of all participants, there were slightly more male WMG athletes (*n* = 1434, 51.3%) than female WMG athletes (*n* = 1359, 48.7%) (Figure 2), and the majority (79.7%) reported having never competed at a previous WMG. A small percentage, approximately one percent, reported having competed in three or more WMG. The majority of WMG participants reported being non-smokers (77.3%) and consumers of one or more standard drinks per week (82.7%). There was no difference in the number of cigarettes smoked per week between genders. Male WMG participants consumed, on average, ~50% more standard alcoholic drinks per week than females (8.0 vs. 5.4, respectively). The five most popular sports participated in by participants were athletics, rowing, swimming, soccer (football) and softball.

### 3.2. Indicators of Obesity

Male WMG athletes were significantly (*p* < 0.001) older (+10.3%) and heavier (+21.3%) than female WMG athletes. With respect to obesity using BMI and waist circumference as indicators, male WMG athletes had a significantly (*p* < 0.001) higher mean BMI (+4.0%) and larger waist circumference (+11.8%) (Table 1). The majority of participants were categorized as normal or overweight, with a similar percentage of WMG participants classified as obese. Regarding waist circumference, both male and female WMG participants were primarily classified (gender specific) as normal; however, there was a difference in high-risk classification with females having twice the percentage as males (22.0% versus 11.1%, respectively) classified as high risk (Table 1). Participants’ mass was found to be significantly and positively correlated to BMI (*p* < 0.001, r = 0.777) as was waist circumference (*p* < 0.001, r = 0.685).

### 3.3. Resting Systolic and Diastolic Blood Pressure

We investigated resting blood pressure (SBP and DBP) and categorized blood pressure according to the new guidelines. Overall, we found the mean SBP for males to be elevated and normotension for females; however, the mean DBP for both genders was normotensive. We found that mean resting blood pressure, both SBP and DBP, was significantly (*p* < 0.001) higher in male versus female WMG participants. The males’ mean SBP was 6.5% higher and DBP was found to be 5.9% higher than female WMG participants. When categorized, the majority of female WMG participants were categorized as normal and males as elevated (49.7% vs. 43.8%, respectively). Collectively, higher percentages (greater than twofold) of male WMG participants were classified as hypertensive for SBP (stages 1, 2 and crisis) versus female WMG participants (36.24% vs. 16.12%).

Regarding overall classification of blood pressure where a SBP ≥ 130 mmHg or a DBP ≥ 80 mmHg, overall, 31.7% of MAs were classified as hypertensive, whereas the NHANES dataset had a higher overall classification of hypertension of 44.5%.

For comparison, the Australian Institute of Health and Welfare in 2019 reported that 33.7% of Australian adults, ages 18 and over, were diagnosed with hypertension (males 36.0%, females 31.4%) [44]. The mean resting BP was 126/77 mmHg for Australian male adults and 119/76 mmHg for Australian female adults. Male WMG participants’ resting SBP was significantly lower (*p* < 0.001, −4.2%) than the Australian comparative group; however, there was no difference (*p* = 0.825) in resting DBP. With respect to DBP, female WMG participants had a significantly lower (*p* < 0.001, −1.7%) SBP and DBP than the Australian general population (*p* < 0.001, −4.5%).

**Figure 3:**
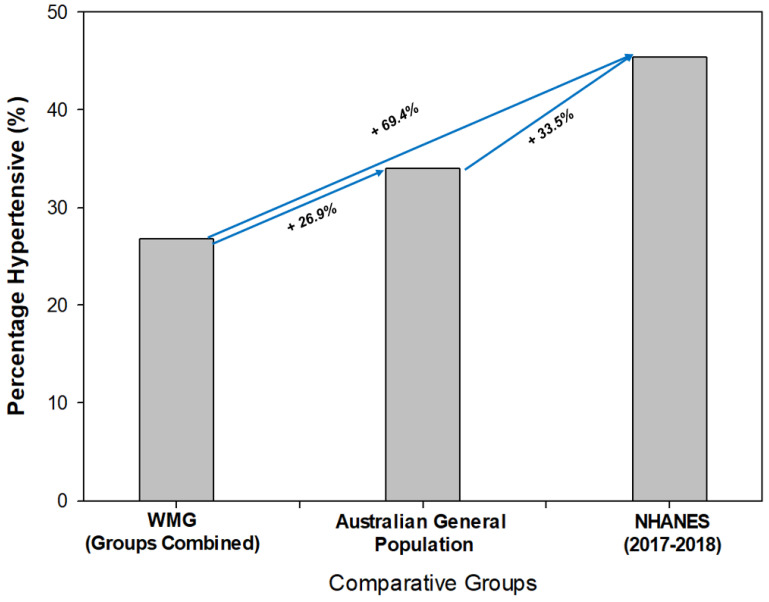
Comparison of prevalence of hypertension between World Master Games (WMG) athletes, Australian general population and National Health and Nutrition Examination Survey data set (NHANES).

When comparing the WMG BP findings to the National Health and Nutrition Examination Survey (NHANES), the WMG participants (groups combined) were significantly lower in their mean SBP (*p* < 0.001, 120.9 mmHg vs. 125.8 mmHg). Regarding DBP, there was no significant difference between the groups (74.9 mmHg vs. 74.4 mmHg, respectively). Additionally, we compared the prevalence of hypertension in our WMG participants to both the general Australian population and the NHANES (2017–2018) findings (Figure 3) [45]. The WMG participants (groups combined) had the lowest prevalence as a percentage compared to both the general Australian population and the NHANES data set from 2017 to 2018. The general population of Australia’s prevalence of hypertension was approximately 26% higher (relative) than WMG, and the NHANES data set was approximately 70% higher (relative) than the WMG participants and higher than the general Australian population (Figure 3). 

A number of our participants reported having hypertension diagnosed by their primary care physician or specialist (*n* = 507; 22% of males, 14% of females) of which 15% were prescribed anti-hypertension medication (18.9% males, 10.9% female WMG participants). When we compared blood pressures of those participants diagnosed with hypertension who were prescribed anti-HTN versus no HTN medication, we found that those on a prescribed anti-HTN medication (groups combined) had a slightly lower SBP (*p* = 0.08, 130.1 ± 11.9 mmHg vs. 132.1 ± 11.9 mmHg) and DBP (*p* = 0.134, 79.5 ± 8.1 mmHg vs. 80.5 ± 9.0 mmHg). Unfortunately, we did not assess compliance with any prescribed medications. Anti-hypertensive medication was reported to be the most prescribed medication (15.0%), followed by anti-lipidemic (8.3%) and non-steroidal anti-inflammatories (7.3%) in our WMG participants. 

We investigated associations between resting blood pressure (systolic and diastolic) and age, mass, BMI and waist circumference. Resting SBP had significant correlations with age (*p* < 0.001, r = 0.211), mass (*p* < 0.001, r = 0.331), BMI (*p* < 0.001, r = 0.275) and waist circumference (*p* < 0.001, r = 0.267). We also identified significant relationships with DBP including age (*p* = 0.004, r = 0.055), mass (*p* < 0.001, r = 0.292), BMI (*p* < 0.001, r = 0.239) and waist circumference (*p* < 0.001, r = 0.252).

**Figure 4:**
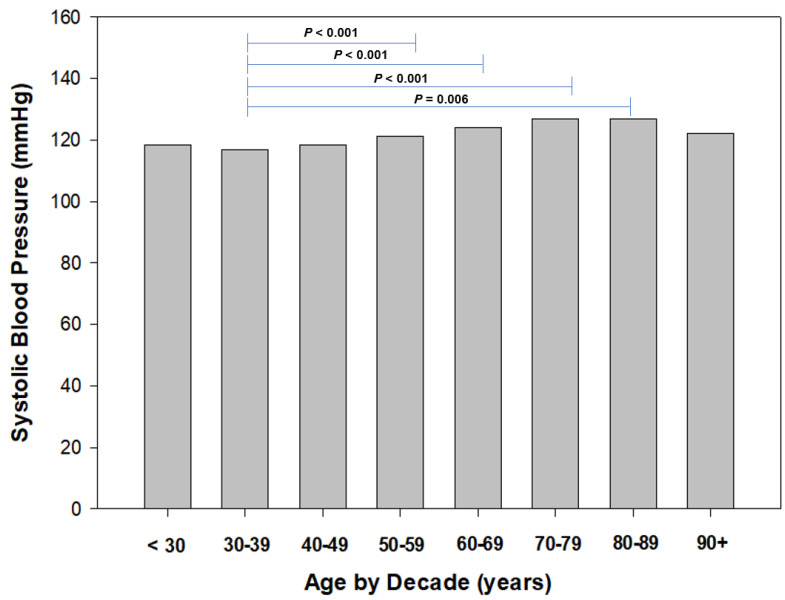
Systolic blood pressure by decade of age.

**Figure 5:**
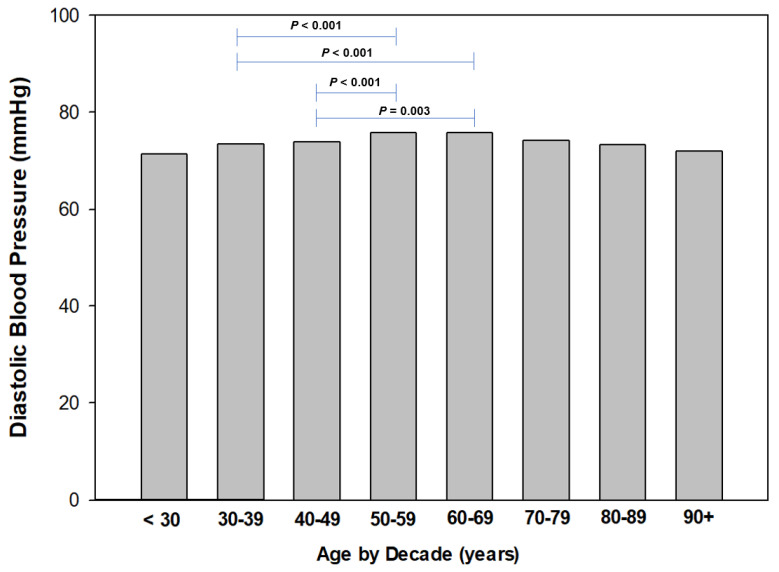
Diastolic blood pressure by decade of age.

To identify age-related trends in resting blood pressure, we stratified age by decade (i.e., <30 years, 30 to 39 years, etc.) for both systolic and diastolic blood pressures (Figure 4 and Figure 5, respectively). Regarding SBP, we found a trend of increased resting SBP from the 30 to 39 age group to 50 to 59 years, and plateauing at the 60 to 69 years group, then declining. We found the mean SBP to be the lowest in the 30- to 39-year-old group and significantly higher (*p* = 0.006 to *p* < 0.001) than other groups (Figure 3). Similarly, we identified significant (*p* = 0.003 to *p* < 0.001) differences in DBP between the 30- to 39-year-old group and 40- to 49-year-old group to other age groups (Figure 4). 

## 4. Discussion

The primary aim of this study was to establish the prevalence of hypertension in WMG participants (male and female). Our secondary aim was to categorize resting systolic and diastolic blood pressure according to the most recent guidelines [5,41] and other outcomes (waist circumference) based upon RACGP guidelines [39]. Further, we also compared our resting SBP and DBP findings to the general Australian population and NHANES data. We successfully surveyed 2793 WMG male and female participants who provided full biometric data of interest to complete this study, the largest sample of WMG participants to date which has investigated resting SBP and DBP and categorized as normotensive, elevated and hypertensive (stage 1, 2 and crisis). There are very limited findings on blood pressure in WMG or master/veteran athletes. Previously in 2011, we reported the prevalence of chronic diseases in Golden Oldies World Rugby Festival (GORF) participants (*n* = 216; mean age 51.2 ± 8.0 years, range 35 to 72 years) [46]. We reclassified resting SBP and DBP to the new classifications [5,41] to compare findings to the current study. Although the focus of the previous study was to determine the prevalence of chronic conditions in GORF participants, we also collected data on SBP and DBP. The prevalence of hypertension in GORF participants, overall, was 18.6% and the most commonly reported health disorder. Regarding SBP, the majority of participants were classified as elevated (37.1%) followed by normotensive (21.9%) and hypertensive (stage 1 (24.8%). No GORF participants were classified as hypertensive stage 3 (crisis). We found a different trend in DBP with the majority (53.3%) of participants classified as hypertensive stage 1, followed by normotensive (37.1%) and hypertension stage 2 (9.5%). Similar to SBP, no GORF participants reported a resting DBP that would classify them as hypertensive stage 3 (crisis). The GORF findings [46] for SBP are similar to our current study where the majority of WMG participants were classified as elevated (39.0%) following by normotensive (34.3%). Our current study did not parallel the GORF findings [46] with regard to DBP where in the current study the majority (55.9%) of participants were classified as normotensive, followed by hypertension stage 1. Additionally, the GORF study identified no participants classified as hypertension stage 3 (crisis), whereas the current study had a small number of participants with the classification (SBP (males = 6, females = 4) and DBP (males = 1, females = 2)). 

With regard to previous studies assessing blood pressure in masters athletes, Hernelahti et al. [33] investigated hypertension in 264 male orienteering runners (mean age 58.5 ± 7.0 years) via survey. They reported 7.6% of their participants being prescribed anti-hypertension medication and 23.9% being advised they had elevated blood pressure. As this study was published prior to the new blood pressure classification guidelines in 1997 and no supplementary data file was available on the actual blood pressures of their participants, we are, unfortunately, unable to make a direct comparison to these findings reported as we cannot re-classify their blood pressure readings. 

Parry-Williams et al. [34] investigated resting blood pressure in 214 male master endurance runners (mean age 51, range 40 to 65 years). They found that over one-half (53.7%) of their MAs had an elevated (classified as abnormal, >130/85 mmHg) blood pressure and approximately one-half (51.4%) were hypertensive (various classifications). However, despite this study being published in 2021, these authors utilized a non-conventional value for “abnormal” blood pressure classification which was ≥130/85 mmHg which under the new classification system [5,41] would be classified as “stage 1 hypertension”. Therefore, making a direct comparison of the Parry-Williams and colleagues [34] findings to ours is not possible.

Our cohort has previously reported SBP and DBP (and categorized) in master athletes; however, a much smaller sample size (*n* = 486) was utilized [32]. In that study, we primarily investigated fasting plasma glucose in masters’ athletes and included blood pressure. We found a similar prevalence of systolic and diastolic hypertension in that study. In Climstein et al. [32] we reported that approximately 45% of the male master athletes reported systolic hypertension, whereas in the current study, we found a much lower percentage (26.8%). With regard to diastolic hypertension, the previous study [32] identified a lower percentage of master athletes with diastolic hypertension (males 44%, females 28%) as compared to our current study (males 54%, females 34%). This is an interesting finding as the participants in the current study had a similar waist circumference and BMI.

Hypertension has been reported previously in athletes in general with the belief that athletes are not immune from developing the disorder [47], and we have seen this in our current group of WMG participants, both male and female. It is interesting that when we investigated our WMG participants who reported being diagnosed with hypertension, and those prescribed an anti-hypertensive medication versus those not prescribed an anti-hypertensive medication, the differences between SBP and DBP were small (SBP 2.0 mmHg, DBP 1.0 mmHg). According to Guzman et al. [48] these differences would represent non-clinically significant differences, as a difference of ≥10 mmHg SBP and ≥5 mmHg DBP are required for a significant clinical benefit in a reduction in cardiovascular risk. This is also supported by the Blood Pressure Lowering Treatment Trialists Collaboration [49] reported from the meta-analyses which indicates that a 5 mmHg reduction of SBP reduced the risk of major cardiovascular events by approximately 10%. Chan [50] discussed minimal clinically important difference with regard to blood pressure. Chan [50] stated that a 2 mmHg difference is not adequate for a clinical difference. Therefore, is it plausible that non-medicated, hypertensive WMG participants realize the same risk reduction benefit as those WMG participants who are medicated with anti-hypertensive therapy with regard to cardiovascular disease risk reduction.

Hypertension in master athletes has been previously reported by Hernelahti et al. [33] who surveyed 286 male orienteering runners in the mid 1970’s on self-reported use of medication for hypertension. They reported approximately eight percent of their MAs were prescribed anti-hypertension medication and 24% of their MAs who were unmedicated stated their blood pressure was elevated. The prevalence of hypertension in the master athletes was one-third that of the age-matched controls (23.5% and 45.4% unmedicated with blood pressure elevated). The authors concluded that long-term participation in endurance training exercise was associated with a low prevalence of hypertension as compared to age-matched, sedentary controls. Unfortunately, Hernelahti and colleagues [33] did not measure blood pressure in their participants due to the survey methodology they utilized. Additionally, the study would have been using the outdated blood pressure classifications (i.e., hypertension ≥ 140/90 mmHg) therefore, if re-evaluated using the new classifications, it is reasonable to expect a higher percentage of those on anti-hypertensive medications.

Given the paucity of studies available on blood pressure in WMG athletes, we chose to identify hypertension in athletes in general. Berge and colleagues [51] conducted a systematic review which included over 138,000 athletes (aged 18 to 40 years). The percentage of hypertension identified was very low at 3.0% in the participants of the studies included in their review. This is well below the identified prevalence of hypertension identified in this study. It has been previously reported that hypertension is more prevalent in male athletes, and this attributed to their increased BMI and use of non-steroidal anti-inflammatory (NSAIDs) medications. In our present study, 3.6% of the male WMG participants and 3.8% of female WMG participants reported taking NSAIDs. 

The effectiveness of exercise versus anti-hypertensive medications has been previously investigated. Naci and colleagues [52] completed a systematic review with meta-analysis of 391 randomized controlled studies (RCTs) that included, collectively, over 10,000 participants. They reported that in the studies included in their review, anti-hypertensive medications resulted in a greater reduction (−3.96 mmHg, 95% CI −5.02–2.91) in baseline SBP compared with the exercise interventions (endurance, dynamic resistance, isometric resistance, and combined endurance and resistance exercise interventions). However, the authors of the study also reported that all types of exercise, including combination endurance plus resistance training, were also effective in lowering baseline SBP. They reported a SBP reduction of 8.96 mmHg when comparing exercise to control. When exercise was compared to common anti-hypertension medications (angiotensin-converting-enzyme inhibitors, Angiotensin II receptor blockers, beta blockers, diuretics), no difference was observed when compared with endurance or resistance training. The authors concluded that the SBP lowering effects of the exercise appeared similar to the commonly prescribed anti-hypertensive medications. Castillo-Garcia et al. [53] also completed a review and meta-analysis of RCTs (391 studies with over 39,000 participants), and they also reported that anti-hypertensive medications and exercise were both similarly effective (anti-hypertension medication −8.78 mmHg versus exercise −8.96 mmHg) in lowering resting SBP. They also identified that a combination of endurance and resistance training resulted in the greatest SBP lowering as compared to endurance, resistance training or isometric training alone. 

The importance of therapeutic adherence to prescribed anti-hypertensive approaches by patients cannot be overlooked, particularly with regard to a polypill strategy. The polypill for hypertension is an anti-hypertensive fixed-dose medication which is a combination of several medications commonly used to treat heart disease and hypertension. The results for the polypill in the treatment of HTN have been shown to be promising and have resulted in improved compliance, improved blood pressure control and cardiovascular outcomes [54,55,56,57] 

Laine et al. [58] assessed hypertension in over 2000 former male elite athletes (mean age 72.3 years) in Sweden. Rather than directly measuring blood pressure or surveying the former athletes, these investigators used an indirect, novel method to deduce the prevalence of hypertension by investigating the athletes’ entitlements to reimbursable anti-hypertensive medication from the Finnish Social Insurance Institution. As this study was published in 2015, they utilized the old blood pressure classifications and reported that 35.2% of their participants were hypertensive. Further, they classified their elite athletes into three groups: endurance, mixed and power. Once participants were stratified into these groups, endurance athletes were identified as having the lowest prevalence (26.6%) followed by mixed (36.2%) and power (38.3%). When investigating the risk of developing hypertension, all three groups were found to have reduced risk compared to age-matched, sedentary controls. Endurance athletes had the lowest odd ratio (OR) for risk for developing hypertension (OR 0.43, 95% CI 0.23–0.80), followed by mixed (OR 0.73, 95% CI 0.49–1.08) and power athletes (OR 0.78, 95% CI 0.48–1.26). The authors also reported that the former athletes who were not prescribed an anti-hypertensive medication had a significantly lower SBP (139.2 mmHg vs. 144.2 mmHg) than the age-matched, sedentary controls. This is a similar trend to what we observed in our WMG participants. 

We found the WMG prevalence of HTN to be lower than the general Australian population and the NHANES data. However, a recent study by Ostchega et al. [42] reported the prevalence (age-adjusted) from the 2017–2018 NHANES study to actually be 44.5%, which is almost twofold that which we observed in our WMG participants (genders combined).

Additionally, we found that SBP plateaued from 60 to 69 years of age onwards, with no further increase in the older age groups. We attribute this finding to the small number of participants aged 80 and older (*n* = 25; males = 21, females = 4). The Australian Institute of Health and Welfare recently reported that hypertension was most prevalent in the age group 85 years and older (47%); we suspect our low participant numbers did not enable us to accurately assess the prevalence of hypertension in our older age groups. In our participants, our older participants (80 years of age +) did not report a SBP > 140 mmH and a DBP > 90 mmHg. Despite the low number of older WMG participants, our findings in this study were quite favorable on these older WMG participants’ resting blood pressures reported. 

### 4.1. Strengths of the Study

The primary strength of our study was the number of WMG participants included. Additionally, the data set contained an almost equal number of male and female WMG participants, whereas a number of the previously published studies only include male master athletes. Also, rather than participants relying on recall, we requested all participants visit their primary care physician or specialist for recent biometric data as opposed to self-monitored readings at home. This is important as Nessler et al. [59] recently identified significant differences in self-monitored blood pressure, where both SBP and DBP were elevated. 

### 4.2. Strengths and Limitations of the Study

The primary strength of our study was the sample size of WMG participants who completed our study, the largest to date investigating BP in MA. Additionally, we only requested participants report their most recent resting SBP and DBP that was attained from their primary care physician or specialist. However, the guidelines for blood pressure assessment (and management) of hypertension [60] allow for blood pressure to be taken by auscultation, automated or 24-h monitors as acceptable to diagnose hypertension. Although it can be assumed that our participants had their resting blood pressure assessed by auscultation or automated, Liu et al. [61] compared the differences of these two methods and found only small (SBP +2.0 mmHg, DBP −6.0 mmHg) differences. Also, we did not require any participants to upload their biometric data due to ethics and privacy concerns. 

There were a number of limitations with this study, as the design was survey; therefore, the risk of sampling bias and response bias exists. We believe we minimized sampling bias in that all participants completing the survey were WMG participants and the total number represents the largest investigated for resting BP to date. Response bias was minimized by our use of primarily closed-ended, drop-down response options. Additional bias could be non-response bias as not all participants completed the survey. A further limitation to the study was that all participants were surveyed as opposed to us measuring resting SBP and DBP. It is highly likely that our participants had their resting blood pressure assessed by either auscultation or automated oscillometric method from their primary care physician or specialist; however, we did not survey the method used to measure blood pressure nor require participants to upload their medical reports. This was an Ethics Committee decision as the medical reports may have contained confidential or personal medical results not relevant to the study. When compared to the NHANES findings [42], their prevalence of HTN was dependent upon if the measurement was via auscultation (44.5%) or oscillometric (i.e., automated, 45.1%). Regardless of the method to assess resting blood pressure in the NHANES study, WMG participants were found to have a much lower prevalence of HTN. Additionally, as the MA participants were aware of the aims of our study, they may have been influenced by the Hawthorne effect and chose to report values that would indicate they were healthier. However, O’Sullivan and colleagues [62] have investigated the Hawthorne effects in health psychology, with no discernable differences noted.

## 5. Conclusions

This study demonstrated the low, but existent, prevalence of hypertension in a large cohort of male and female WMG participants. The prevalence of hypertension in WMG participants was lower than both the general Australian population and the NHANES data set. Our findings are generalizable to those that we surveyed in this study. We believe the health metric of resting blood pressure adds credence to the notion that master athletes represent a model of successful aging. Future research should focus upon longitudinal changes in resting blood pressure in WMG participants throughout their lifetime.

## Figures and Tables

**Table 1:** Demographics and health biometric outcome variables.

	Group	Males	Females
	(*n* = 2793)	(*n* = 1434)	(*n* = 1359)
•Age (yr)	53.3 ± 9.9	55.8 ± 9.8	50.6 ± 9.4 * < 0.001
	(52.9–53.7)	(55.3–56.3)	(50.1–51.1)
•Smoking status			
-Non-smoker (%)	77.3	78.5	75.9
-Ex-smoker (%)	19.9	20	19.9
-Smoker (%)	2.8	1.5	4.2
•Drinking status			
-Non-drinker (%)	16.4	16	16.7
-Ex-drinker (%)	0.8	1.3	0.6
-Drinker (%)	82.7	82.7	82.7
-Drinks/wk (n)	6.8 ± 6.4	8.0 ± 7.2	5.3 ± 5.1
•Mass (kg)	76.7 ± 14.9	83.8 ± 12.9	69.2 ± 13.0 * < 0.001
	(76.2–77.3)	(83.1–84.5)	(68.5–69.9)
•BMI (kg/m^2^)	25.7 ± 4.2	26.2 ± 3.6	25.1 ± 4.6
	(25.5–25.4)	(26.0–26.4)	(24.8–25.3)
•BMI Classification			
-Underweight (%)	0.08	0.1	1.5
-Normal (%)	49.8	41.9	56.8
-Overweight (%)	36.8	44.6	27.6
-Obese (%)	12.5	12.6	12.1
•Waist circumference	85.4 ± 11.9	89.8 ± 10.4	80.0 ± 11.5 * < 0.001
classification	(84.9–85.9)	(89.2–90.4)	(79.3–80.7)
-Normal (%)	61.7	68.9	52.7
-Increased risk (%)	22.4	20	25.3
-High risk (%)	15.9	11.1	22
•Resting SBP (mmHg)	120.9 ± 12.6	124.6 ± 11.5	117.0 ± 12.5 * < 0.001
	(120.4–121.4)	(124.0–125.2)	(116.4–117.7)
•SBP Classification			
-Normal (%)	34.3	19.9	49.7
-Elevated (%)	39.0	43.8	34.1
-HTN stage 1 (%)	17.6	24.3	10.6
-HTN stage 2 (%)	8.8	11.9	5.5
•HTN crisis (%)	0.4	0.04	0.02
•Resting DBP (mmHg)	74.9 ± 9.2	77.1 ± 8.4	72.7 ± 9.5 * < 0.001
Classification	(74.6–75.3)	(76.6–77.5)	(72.2–73.2)
-Normal (%)	55.9	46.2	66.1
-HTN stage 1 (%)	37.5	45.7	29
-HTN stage 2 (%)	6.5	8	4.8
-HTN crisis (%)	<0.001	<0.001	0.001

Values are mean ± SD (95% confidence interval) and percent (%, where indicated). Where: * = *p* < 0.001.

## Data Availability

Data is available upon request from the investigators.

## Supplementary Materials

The following supporting information can be downloaded at: https://www.mdpi.com/article/10.3390/sports11040085/s1, STROBE Statement—checklist of items that should be included in reports of observational studies.
