# Supplementary material for: Resting Blood Pressure in Master Athletes: Immune from Hypertension?
Source: Sports (Basel). 2023 Apr 18;11(4):85. doi: 10.3390/sports11040085 (PMC10144796; doi:10.3390/sports11040085)
Supplement: Supplementary file 1 [file sports-11-00085-s001.zip › sports-2295407-supplementary.pdf]

Resting Blood Pressure in Master Athletes: Immune from Hypertension?

STROBE Statement—checklist of items that should be included in reports of observational studies

	Item No.	Recommendation	Page No.	Relevant lines from manuscript
**Title and abstract**	1	(*a*) Indicate the study's design with a commonly used term in the title or the abstract		Abstract 20
		(*b*) Provide in the abstract an informative and balanced summary of what was done and what was found		18-32
**Introduction**				
Background/rationale	2	Explain the scientific background and rationale for the investigation being reported		37-124, rationale 111-119
Objectives	3	State specific objectives, including any prespecified hypotheses		119-124
**Methods**				125-201
Study design	4	Present key elements of study design early in the paper		Ethics 126-135<br>Survey design 136-142<br>Participants and survey 143-159<br>Biometric classifications 162-181<br>Comparative data 183-188
Setting	5	Describe the setting, locations, and relevant dates, including periods of recruitment, exposure, follow-up, and data collection		Survey was open 9/2009 and closed 12/2009 135-137
Participants	6	(*a*) *Cohort study*—Give the eligibility criteria, and the sources and methods of selection of participants. Describe methods of follow-up<br>*Case-control study*—Give the eligibility criteria, and the sources and methods of case ascertainment and control selection. Give the rationale for the choice of cases and controls<br>*Cross-sectional study*—Give the eligibility criteria, and the sources and methods of selection of participants		Eligibility 143-145<br>Selection 137-138 (convenience sample)<br>no follow-up<br>184-188, ABS data selected as represents general Australia population, NHANES data selected an 2nd comparative group
		(*b*) *Cohort study*—For matched studies, give matching criteria and number of exposed and unexposed		NA

		Case-control study—For matched studies, give matching criteria and the number of controls per case	
Variables	7	Clearly define all outcomes, exposures, predictors, potential confounders, and effect modifiers. Give diagnostic criteria, if applicable	Biometrics classifications 163-181 as per RACGP classifications
Data sources/ measurement	8*	For each variable of interest, give sources of data and details of methods of assessment (measurement). Describe comparability of assessment methods if there is more than one group	Biometrics classifications 163-181 as per RACGP classifications
Bias	9	Describe any efforts to address potential sources of bias	Bias is addressed in the limitations but includes sampling bias, response bias, non-response -bias
Study size	10	Explain how the study size was arrived at	NA, a convenience sample was used 138-140

Continued on next page

Quantitative variables	11	Explain how quantitative variables were handled in the analyses. If applicable, describe which groupings were chosen and why	All quantitative variables were standard clinical measures with classifications used according to the RACGP 164-183
Statistical methods	12	(*a*) Describe all statistical methods, including those used to control for confounding	192-203
		(*b*) Describe any methods used to examine subgroups and interactions	WMG participants were sub grouped by gender and by decade of age
		(*c*) Explain how missing data were addressed	
		(*d*) *Cohort study*—If applicable, explain how loss to follow-up was addressed<br>*Case-control study*—If applicable, explain how matching of cases and controls was addressed<br>*Cross-sectional study*—If applicable, describe analytical methods taking account of sampling strategy	NA<br>NA<br>Sampling strategy was to sample all WMG participants who registered with a valid email address.127-144
		(*e*) Describe any sensitivity analyses	NA
**Results**			
Participants	13*	(a) Report numbers of individuals at each stage of study—eg numbers potentially eligible, examined for eligibility, confirmed eligible, included in the study, completing follow-up, and analysed	Fig 1 CONSORT diagram
		(b) Give reasons for non-participation at each stage	Fig 1 CONSORT diagram
		(c) Consider use of a flow diagram	Fig 1 CONSORT diagram
Descriptive data	14*	(a) Give characteristics of study participants (eg demographic, clinical, social) and information on exposures and potential confounders	Table 1, age, smoking status, drinking status, ect
		(b) Indicate number of participants with missing data for each variable of interest	As per Methods, only participants with both SBP and DBP were included in this study. Fig 1 shown that a total of 8,070 participants completed our study, however only 2,793 submitted BP results.
		(c) *Cohort study*—Summarise follow-up time (eg, average and total amount)	NA
Outcome data	15*	*Cohort study*—Report numbers of outcome events or summary measures over time	*NA*
		Case-control study—Report numbers in each exposure category, or summary measures of exposure	*NA*

		Cross-sectional study—Report numbers of outcome events or summary measures	*See Table 1, Figures 3,4 and 5 and Results 204- 314*
Main results	16	(*a*) Give unadjusted estimates and, if applicable, confounder-adjusted estimates and their precision (eg, 95% confidence interval). Make clear which confounders were adjusted for and why they were included	95%Cis listed for select variables in Table 1
		(*b*) Report category boundaries when continuous variables were categorized	RACGP classifications 164-184
		(*c*) If relevant, consider translating estimates of relative risk into absolute risk for a meaningful time period	NA

Continued on next page

Other analyses	17	Report other analyses done—eg analyses of subgroups and interactions, and sensitivity analyses	204-314, T-tests, ANOVA, correlations
**Discussion**			
Key results	18	Summarise key results with reference to study objectives	316-324
Limitations	19	Discuss limitations of the study, taking into account sources of potential bias or imprecision. Discuss both direction and magnitude of any potential bias	Section 4.2 Strengths and Limitation 474-501
Interpretation	20	Give a cautious overall interpretation of results considering objectives, limitations, multiplicity of analyses, results from similar studies, and other relevant evidence	204-501
Generalisability	21	Discuss the generalisability (external validity) of the study results	508
**Other information**			
Funding	22	Give the source of funding and the role of the funders for the present study and, if applicable, for the original study on which the present article is based	No funding was provided for this study. 517

*Give information separately for cases and controls in case-control studies and, if applicable, for exposed and unexposed groups in cohort and cross-sectional studies.

Note: An Explanation and Elaboration article discusses each checklist item and gives methodological background and published examples of transparent reporting. The STROBE checklist is best used in conjunction with this article (freely available on the Web sites of PLoS Medicine at http://www.plosmedicine.org/, Annals of Internal Medicine at http://www.annals.org/, and Epidemiology at http://www.epidem.com/). Information on the STROBE Initiative is available at www.strobe-statement.org.
